# Degenerated oocyte in the cohort adversely affects IVF outcome

**DOI:** 10.1186/s13048-020-00708-6

**Published:** 2020-09-17

**Authors:** Yuval Atzmon, Mediea Michaeli, Diana Poltov, Nechami Rotfarb, Oshrit Lebovitz, Nardin Aslih, Einat Shalom-Paz

**Affiliations:** IVF Unit, Department of Obstetrics and Gynecology, Hillel-Yaffe Medical Center, Hadera, Israel; affiliated with the Ruth and Bruce Rappaport School of Medicine, The Technion – Israel Institute of Technology, Haifa, Israel

**Keywords:** Degenerated oocyte, Embryo morphokinetics, Ovum pick-up, Top-quality embryo, Aspiration needle

## Abstract

The presence of Degenerated Oocyte (DEG) was mostly described after intracytoplasmic sperm injection (ICSI), with fewer reports on DEG at the time of ovum pick-up (OPU). This study aims to assess morphokinetics of embryos cultured in a time-lapse incubator and compare cohorts with and without DEG at OPU. In a retrospective cohort study from January 1, 2016 until September 31, 2017 a total of 399 IVF/ICSI cycles and 2980 embryos were evaluated. In 81 of 399 cycles at least one DEG oocyte was observed at the time of OPU. The remaining 318 cycles with no DEG oocyte were compared as a control group. In the DEG group, significantly more oocytes were collected per patient (12.9 ± 7.2 vs. 10.1 ± 6.1. *P* < 0.001). Fertilization rate, pregnancy and clinical pregnancy rates were comparable between the two groups, however, the morphokinetics and developmental scores of the embryos were significantly worse in the DEG group, (KID 3.4 ± 1.6 vs. 3.2 ± 1.6 *P* = 0.002 and ESHRE 1.5 ± 1.1 vs. 1.4 ± 1.0 *P* = 0.046). Significantly more patients achieved top-quality embryos in the NON DEG group (58.8% vs. 53.0%, *P* = 0.03), however, comparable delivery rate was achieved in both groups. In the DEG group, the frequency of DEG oocyte per cycle was negatively correlated with pregnancy rate. GnRH agonist protocol and the 17-20G needle used for OPU were significant predictors for the presence of DEG oocyte at OPU. In conclusions DEG oocyte may negatively affect IVF outcome, however, younger patients, and significantly more oocytes collected in the DEG group compensate for the IVF results.

## Introduction

**Fig. 1:**
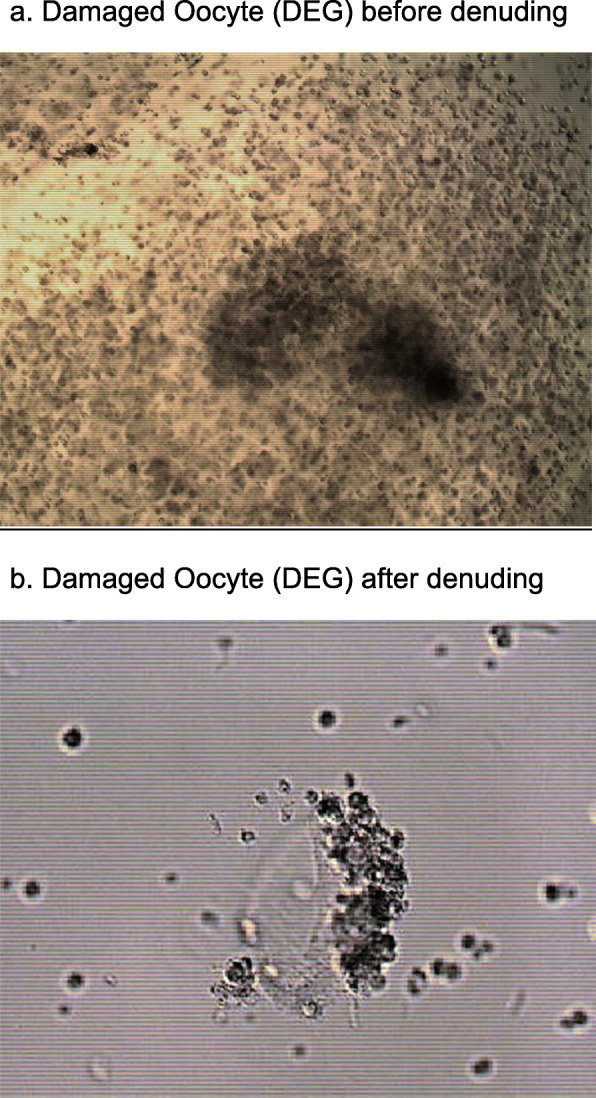
A degenerated oocyte (DEG) at ovum pick-up (OPU)

The success rate of in vitro fertilization (IVF) depends on several parameters, of which good quality oocytes is the most important [1]. A degenerated (DEG) oocyte is described as an empty zona pellucida (EZP) or damaged oocyte (fragments of oocytes) within the zona pellucida [2]. These oocytes can be seen at the time of ovum pick-up (OPU) or after intracytoplasmic sperm injection (ICSI) (Fig. 1). To the best of our knowledge the presence of DEG oocytes immediately after OPU has not been previously studied as itis usually not reported by embryology labs. Oocyte quality before ICSI is very important and affects treatment outcomes. The presence of EZP or DEG oocyte at OPU before ICSI correlates with the quality of the entire oocyte cohort [3–6]. Studies that examined the presence of EZP oocyte at OPU, found worse quality of oocytes collected in the same cycle, lower fertilization, poor embryo formation, and low pregnancy rates [2, 7]. Cinar et al. [2] reported worse performance of oocyte cohort when EZP oocyte were present in the aspirated group. Our study focused on DEG oocyte at the time immediately after OPU.

Currently, an automated time-lapse incubator allows for continuous and objective evaluation of fertilized oocytes and early embryo developmental morphokinetics [8], using the Known Implantation Data (KID) score which assigns morphokinetic parameters from 1 to 5 to estimate embryo viability and implantation potential [9]. Using embryo viewer software, kinetic markers are used in accordance to specific guidelines for: pronuclei (PN) assessment, PN fading, time (t) to 2, 3, 4, 5 and 8 cells. Additional kinetic markers and an Alfa ESHRE score, as well as the common morphology grade are also used for evaluation of the embryos [10].

The presence of DEG oocyte in the cohort of aspirated oocytes reduces the number of oocytes available for fertilization, whether they affect cycle outcomes is still questionable. To date, no study has evaluated the correlation between the presence of DEG oocyte at OPU and embryo morphokinetics. This study assessed morphokinetics of embryos cultured in a time-lapse incubator and compared cohorts with and without DEG oocytes.

## Material and methods

This retrospective cohort study was conducted in a single reproductive centre. Records of all patients and their embryos were collected. The information of all embryos cultured in a time-lapse incubator from January 1, 2016 until September 31, 2017 were evaluated. To reflect the broad range of patients typically encountered in clinical practice, no inclusion/exclusion criteria were applied regarding baseline characteristics apart from the fact that no testicular sperm cycles were included. Cycles in which transfers were cancelled due to endometrial polyps, premature progesterone elevation and the use of donor oocyte were not included in the study. Institutional Review Board approval was obtained for this retrospective study.

The treatment protocol, type and doses of gonadotropins were prescribed on a case-by-case basis, based on patient characteristics and clinician preferences and judgment. The initial dose of gonadotropin was individualized for each patient according to age, basal follicle stimulating hormone (FSH) levels, antral follicle count, body mass index (BMI), and previous response to ovarian stimulation. Three main protocols were included in the study: long agonist, short flare and antagonist. Patients underwent controlled ovarian stimulation by recombinant follicle stimulating hormone (rFSH) alone (Gonal-F, Merck-Serono; or Puregon, MSD); highly purified human menopausal gonadotropin (HPhMG) alone (Menopur, Ferring Pharmaceutical); or rFSH combined with HP-hMG. All treatments were conducted as previously described [11–14]. Estrogen and progesterone levels were measured at every follow-up visit, including the day of human chorionic gonadotropin (hCG) (Ovitrelle Merck-Serono) administration, before egg retrieval. hCG was administered for final maturation of oocytes when at least three mature (> 17 mm) follicles were identified by transvaginal scan, combined with appropriate peripheral serum E2 levels. Oocytes were aspirated approximately 36 h after hCG injection. For luteal phase support, patients received 300 mg micronized progesterone (Endometrin®, Ferring, Israel) in three divided doses daily. Two different aspiration needles were routinely used 17G/35mm (Cook Medical™, Bloomington, IN, USA) and 20-17G/35mm (Sense™, Vitrolife Sweden AB, Gothenburg, Sweden).

After oocyte retrieval, IVF or ICSI was performed. After ICSI, the injected oocytes were placed on EmbryoSlides with one-step medium with SPS (SAGE; Origio) and incubated in the automated time-lapse EmbryoScope™ (Unisense FertiliTech, Aarhus, Denmark) up to 5 days with 5.8% CO2 at 37.0 °C and 5% O2. Using embryo viewer software Images of each embryo were acquired every 10 min in 7 focal planes, starting from the second polar body extraction up to 120 h after fertilization, to determine the exact timing of cell divisions [8]. They received a Known Implantation Data score (KID) [9], and Alfa ESHRE score, as well as the common morphology grade [10]. A maximum of two embryos were transferred on day 3 or one on day 5 of embryo development. The remaining top-quality embryos were vitrified and used in the next frozen embryo transfer, if no pregnancy was achieved in the fresh cycle. Embryo quality was evaluated as well, on the day of transfer according to number of cells, symmetry, granularity, type, percentage of fragmentation, presence of multinucleate blastomers, and degree of compaction, as previously described [15]. A top-quality embryo included the following parameters: 4–5 cells on day 2 or 3; > 6 equal-sized blastomeres and ≤ 20% fragmentation; no multinucleate cells and KID score and Alfa ESHRE score of 5,3 or 5,2 or 4,3 or 4,2, respectively.

Data collection included baseline parameters (age, parity, BMI, number of previous IVF/ICSI cycles, basal FSH), cycle characteristics (length of follicular phase, amount of gonadotropins used, endometrial thickness and estradiol levels on day of hCG administration) and cycle outcomes (number of oocytes retrieved, fertilization and cleavage rates, number of top-quality embryos, and whether clinical pregnancy occurred).

β-hCG test was measured 14 days after embryo transfer, and the clinical pregnancy and implantation rates were confirmed when a gestational sac with fetal heart beat was visible by ultrasound examination after 6 weeks of pregnancy. Demographic data, treatment information and results, and pregnancy outcome were recorded and followed until delivery.

### Statistical analysis

Statistical analysis was performed using the SPSS software package (SPSS Inc., Chicago, IL). We used Shapiro Wilks test to evaluate the distribution of the data. Comparisons were analyzed using Student’s t test or Mann-Whitney U test, when appropriate. Proportions were compared using Chi-square test or Fisher exact test. *P*-value less than 0.05 was considered significant. We used multivariate logistic regression analysis to test all possible factors that may contribute to the occurrence or presence of DEG oocyte in the cohort of aspirated oocytes, and thus might have influenced clinical results.

## Results

A total of 399 IVF/ICSI cycles and 2980 embryos were evaluated. In 81 of 399 cycles at least one DEG oocyte was observed at the time of OPU (DEG GROUP). The remaining 318 cycles without presence of DEG oocyte at OPU (NON DEG GROUP) were compared as a control group. Table 1 presents baseline patient characteristics of both groups.

**Table 1:** Patient characteristics

Characteristic	Non DEG group (*n* = 318)	DEG group (*n* = 81)	*P*-value
Age (years) (mean ± STDV)	35.1 ± 5.9	34.4 ± 5.8	NS
BMI (mean ± STDV)	25.1 ± 5.5	25.9 ± 5.6	NS
Etiology of infertility			
Age/Unexplained/single	117 (36.8%)	34 (45.7%)	NS
PCOS/Anovulation	13 (4.1%)	4 (4.9%)	NS
Male factor	111 (34.9%)	31 (38.3%)	NS
Mechanical/Endometriosis	40 (12.6%)	5 (6.2%)	NS
Combined	37 (11.6%)	4 (4.9%)	NS
LH	5.9 ± 2.7	5.8 ± 2.6	NS
FSH	8.5 ± 2.7	7.6 ± 2.7	NS
E2	70.3 ± 78.9; 44 [29.5–71.5]	72.7 ± 65.0; 50 [39.5–89.5]	NS

*DEG oocyte* Degenerated oocyte, *BMI* Body mass index, *PCOS* Polycystic ovary syndrome, *LH* Luteinizing hormone, *FSH* Follicle stimulating hormone

Tables 2 and 3 show treatment parameters and outcomes comparing the DEG and NON DEG groups. In the DEG group, significantly more oocytes were collected per patient (12.9 ± 7.2 vs. 10.1 ± 6.1, *P* < 0.001) and a trend towards a higher number of mature oocytes was seen (7.3 ± 4.4 vs. 8.4 ± 4.9 *P* = 0.063). A significantly higher serum estradiol level on hCG trigger day (1638 ± 798 pg/ml vs. 1990 ± 1304, *P* = 0.002) was found in the DEG GROUP. No difference was found in fertilization rates. Morphokinetics and developmental scores of the embryos were significantly worse in the DEG GROUP, (KID 3.4 ± 1.6 vs. 3.2 ± 1.6 P = 0.002 and ESHRE 1.5 ± 1.1 vs. 1.4 ± 1.0 *P* = 0.046). Importantly, significantly more patients achieved top-quality embryos in the NON DEG group (58.8% vs. 53.0%, *P* = 0.03). Pregnancy rate and clinical pregnancy rate were not affected and were comparable between the two groups (Table 2).

**Table 2:** Cycle characteristics and outcomes

Characteristic	Non DEG group (*n* = 318)	DEG group (*n* = 81)	*P*-value
Estradiol on hCG trigger day (pgr/dl)	1638 ± 798	1990 ± 1304	0.002
Progesterone level at hCG trigger day	0.67 ± 0.45	0.74 ± 0.47	NS
Endometrium (mm)	9.7 ± 2.3	9.6 ± 2.2	NS
Duration of treatment (days)	9.9 ± 2.6	10.5 ± 2.6	NS
Protocol Number/total cycles (%)			
Long Protocol	51 (16.2)	21 (27.6)	0.03
Flare/Short agonist	26 (8.3)	11 (14.5)	NS
Antagonist	233 (74)	44 (57.9)	0.007
Modified natural cycle	4 (1.3%)	0	NS
**Treatment outcome**			
Number of Oocyte collected	10.1 ± 6.1	12.9 ± 7.2	P < 0.001
M2	7.3 ± 4.4	8.4 ± 4.9	P = 0.063
2PN	5.8 ± 3.8	6.2 ± 3.9	NS
KID	3.4 ± 1.6	3.2 ± 1.6	P = 0.002
ESHRE	1.5 ± 1.1	1.4 ± 1.0	P = 0.046
KID =4 + 5 (Top quality embryo)	1174 (58.8%)	314 (53.0%)	*P* = 0.012
ESHRE 2 + 3 (Top quality embryo)	1041 (52.2%)	282 (47.6%)	*P* = 0.049
TOP quality embryo (combined score of- 5,3/5,2/4,3/4,2)	965/1995 (48.4%)	257/593 (43.3%)	*P* = 0.031
Chemical pregnancy/cycle	105/318 (38.5%)	32 (39.5%)	NS
Clinical pregnancy/cycle	105/318 (38.5%)	29 (35.8%)	NS

*DEG* Degenerated oocyte, *hCG* Human chorionic gonadotropin

**Table 3:** Multivariate analysis to predict DEG oocytes in the cohort

Woman’s age	Odds ratio	95% CI		***P***-value
	0.980	0.932	1.029	0.415
BMI	0.967	0.920	1.016	0.188
Long protocol	2.604	1.344	5.042	0.005
Flare protocol	3.250	1.389	7.605	0.007
Needle type	2.035	1.146	3.613	0.015

*DEG oocyte* Degenerated oocyte, *BMI* Body mass index

A multivariate analysis was conducted to test all possible factors that may contribute to the presence of DEG oocyte in the cohort (Table 3). Maternal age, BMI, simulation protocols and type of needle used for OPU were included in the analysis. Significant predictors for the presence of a DEG oocyte at OPU were Stimulation protocols using decapeptyl (Flare and Long) compared with Antagonist protocol (Flare protocol: odds ratio (OR = 3.25, 95%CI-1.39-7.60, *P* = 0.007) and Long down regulation protocol: OR = 2.60, 95%CI = 1.34–5.04, *P* = 0.005). The chance for DEG was higher when OPU was conducted with 17-20G needle compared with the 17G needle (OR = 2.035, 95%CI = 1.115–3.61, *P* = 0.015).

In analyses of the DEG group for the prevalence of DEG oocytes in the cohort at OPU, we found that a lower proportion of DEG in the cohort resulted in a higher pregnancy rate (OR = 0.11, 95%CI 0.02–0.56, *P* = 0.008). The ratio of DEG oocytes per cycle was negatively correlated with pregnancy rate. In the group of DEG oocytes, we found that OPU conducted with 17G needle resulted in a higher pregnancy rate as compared with 17-20G needle (47.8% vs. 23.8%; *P* < 0.0001).

## Discussion

The current study evaluated the effect of degenerated oocytes (DEG) found at OPU on clinical outcomes, including cycle outcome and embryo morphokinetics comparing the presence and absence of DEG oocyte in the cohort of aspirated oocytes. This is the largest study to account and evaluate the presence of DEG at OPU. Our results revealed that the impact of the stimulation protocol and the size of needle used for OPU had significant influence on presence of DEG oocyte in the cohort at OPU.

Based on previous studies, the presence of DEG in the cohort of aspirated oocytes can be the result of physical and mechanical forces contributed by needle bevel, aspiration vacuum pressure [16], technique of OPU including scraping of the follicle and needle type [15, 17]. Additional factors are the intrinsic oocyte quality, which can be influenced by infertility cause, obesity and stimulation protocol [2, 5, 18–20].

The cause of DEG oocyte and its impact at OPU are only minimally discussed in the literature. Cohen et al. [16] showed that manual aspiration by a syringe resulted in a higher rate of damaged oocytes compared with mechanical aspiration by pump [16]. Oride et al. [7] hypothesized that the mechanical forces during pick up caused oocyte damage at OPU and had an impact on cycle outcome. They demonstrated that cycles with higher number of oocytes significantly correlated with increased presence of DEG oocytes, and the fertilization rate and cleavage rate of those cycles were significantly lower [7]. Cinar et al. [2] evaluated the impact of the ratio of DEG oocyte of the total aspirated oocytes per pick-up. They found that the ratio (DEG/total oocyte) increased when more oocytes were collected per cycle. This study, in agreement with our results, reported a negative correlation between the DEG ratio to fertilization rate and cleavage rate. Pregnancy rate was highest when no DEG were collected [2].

Our cohort included a group of 81 cycles (20%) in which at least one DEG oocyte was found (Fig. 1). Possibly a causative correlation exists between the presence of DEG oocyte and the sheer stress applied along the needle. Animal models demonstrated the effect of aspiration pressure and needle size on damaged oocytes [21, 22]. It is well established that according to Hagen-Poiseuille’s law [23], at the same pressure, the flow in a small gauge needle is slower than in larger gauge. Meaning that the Laminar flow within a needle shows a parabolic distribution due to different velocities of the fluid along the needle. Due to suction forces along the shaft, fluid moves slower close to the inner wall of the needle and faster in its center. In addition, the oocyte size is smaller than the needle diameter, and due to different shear stresses, the oocyte comlex may be bounced inside the lumen. It was shown that higher pressure and thicker needles can damage the cumulus-oocyte-complex and was suggested to have great effect on oocyte and embryo quality and reduce the blastulation rate [16, 17, 21, 24].

Human studies assessing oocyte quality report conflicting data regarding the impact of DEG oocyte on cycle outcome [3, 4, 19, 20, 25–29]. Lazzaroni-Tealdi et al. [6] use oocyte scoring to provide useful information on embryo quality and showed that oocyte score provided significantly greater predictive value for clinical pregnancy. Shi et al. [19] reported significantly lower fertilization rate, poorer embryo quality, implantation and clinical pregnancy rate in the group of patients that had at least one damaged zona pellucida in the cohort. We assumed that the same force that caused the DEG oocyte might influence the rest of the cohort and lead to the reduced fertilization, embryo quality and cycle results. We found a significant difference in embryo morphokinetics, with poorer embryo scoring in the DEG group, but without significant differences in implantation and clinical pregnancy rates. The comparable outcome between the groups might be due to compensation by a higher number of collected oocytes or the fact that eventually, a top-quality embryo was transferred.

Our study found that the needles used for OPU and the treatment protocol were associated with DEG oocyte at OPU. We used two different types of needles for oocyte aspiration, a 17G needle or a changing diameter 17-20G needle. The vacuum pressure is different for each needle, 140 mmHg for 17G and 120 mmHg for the 17-20G (manufactory recommendation). In multivariate analysis, we found that using 17-20G needle was significantly correlated with a higher number of DEG oocytes at OPU and a significantly worse morphokinetics score. However, in agreement with Wikland et al. [30] our study did not show any difference between the two needles in pregnancy rates.

Stimulation protocol was also found to impact the presence of DEG. Protocols which used GnRH agonist including long down regulation protocol and flare protocol had twice and three times higher risk of DEG oocyte, respectively, as compared with antagonist protocol. In contrast to our results, Cinar et al. [2] reported that more damaged oocytes was correlated with the use of GnRH antagonist protocol.

The limitations of our study are inherent to its retrospective nature and to the patient treatment protocol and the fact that this was a heterogenic group with different causes of infertility. About 70% of our patients start with antagonist protocol, only after failure of the antagonist protocol, we change the protocol to long down regulation or flare.

The strengths of our study are that it is one of the largest to report DEG oocyte at OPU, the impact on oocyte performance and the follow-up until pregnancy.

**Fig. 2:**
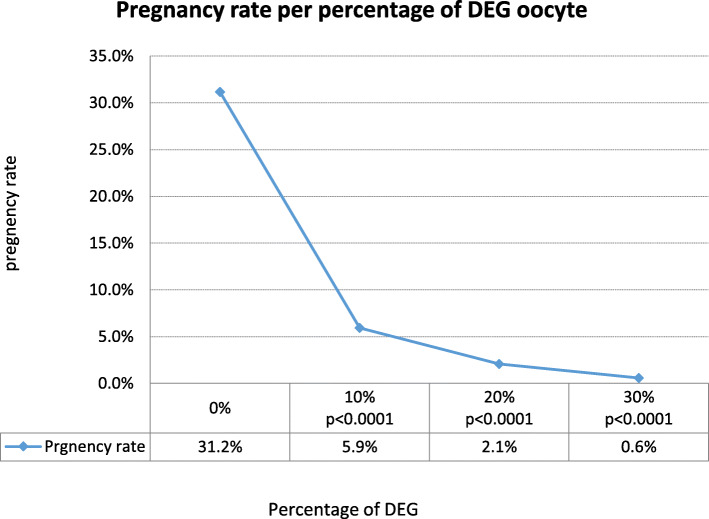
The impact of the prevalence of DEG oocyte on pregnancy outcome

In conclusion, we found a negative correlation between the prevalence of DEG oocyte in the cohort to pregnancy rate. As the percentage of DEG oocytes increased, the pregnancy rate significantly decreased (Fig. 2). Another important finding was the association between the number of aspirated oocytes and the percentage of DEG. Taken together, these findings may reflect on the impact of the aspiration forces along the needle or the type of protocol used in the cohort of the DEG group. Further studies are needed to assess the influence of the protocols, the medication used and the needle type on DEG oocytes.

## Data Availability

Yes
